# The *malQ* gene is essential for starch metabolism in *Streptococcus mutans*

**DOI:** 10.3402/jom.v5i0.21285

**Published:** 2013-08-06

**Authors:** Yutaka Sato, Kazuko Okamoto-Shibayama, Toshifumi Azuma

**Affiliations:** 1 Department of Biochemistry, Tokyo Dental College, Chiba, Japan; 2 Department of Microbiology, Tokyo Dental College, Chiba, Japan; 3 Oral Health Science Centre, Tokyo Dental College, Chiba, Japan

**Keywords:** malR, glgP, maltose, maltooligosaccharide, glucanotransferase, phosphorylase, glucose-releasing activity

## Abstract

**Background:**

The *malQ* and *glgP* genes, respectively, annotated as putative 4-α-glucanotransferase and putative glycogen phosphorylase are located with a 29 nucleotide overlap on the *Streptococcus mutans* genome. We found that the *glgP* gene of this organism was induced with maltose, and the gene likely constituted an operon with the upstream gene *malQ*. This putative operon was negatively regulated with the *malR* gene located upstream from the *malQ* gene and a MalR-binding consensus sequence was found upstream of the *malQ* gene. *S. mutans* is not able to catabolize starch. However, this organism utilizes maltose degraded from starch in the presence of saliva amylase. Therefore, we hypothesized that the MalQ/GlgP system may participate in the metabolism of starch-degradation products.

**Methods:**

A DNA fragment amplified from the *malQ* or *glgP* gene overexpressed His-tagged proteins with the plasmid pBAD/HisA. *S. mutans malQ* and/or *glgP* mutants were also constructed. Purified proteins were assayed for glucose-releasing and phosphorylase activities with appropriate buffers containing maltose, maltotriose, maltodextrin, or amylodextrin as a substrate, and were photometrically assayed with a glucose-6-phosphate dehydrogenase–NADP system.

**Results:**

Purified MalQ protein released glucose from maltose and maltotriose but did not from either maltodextrin or amylodextrin. The purified GlgP protein did not exhibit a phosphorylase reaction with maltose or maltotriose but generated glucose-1-phosphate from maltodextrin and amylodextrin. However, the GlgP protein released glucose-1-phosphate from maltose and maltotriose in the presence of the MalQ protein. In addition, the MalQ enzyme activity with maltose released not only glucose but also produced maltooligosaccharides as substrates for the GlgP protein.

**Conclusion:**

These results suggest that the *malQ* gene encodes 4-α-glucanotransferase but not α-1,4-glucosidase activity. The *malQ* mutant could not grow in the presence of maltose as a carbon source, which suggests that the *malQ* gene is essential for the utilization of starch-degradation products.

*Streptococcus mutans* is a major etiologic agent of human dental caries (1). Although some strains of this organism have been recently reported as agents of infective endocarditis (2) as well as aggravating agents of hemorrhagic stroke (3) and ulcerative colitis (4) when they entered the blood streams of animals, the natural habitat of *S. mutans* was an oral biofilm called dental plaque. Here, *S. mutans* is subjected to continual cycles of abundance and depletion (so-called ‘feast’ and ‘famine’) (5) with respect to carbohydrate energy sources. *S. mutans* accumulates intracellular polysaccharides as energy reserve materials similar to glycogen and starch in animals and plants, respectively (6). Therefore, *S. mutans* harbors genes encoding intracellular polysaccharide synthesis and degradation enzymes in its genome. The *malQ* and *glgP* genes respectively annotated as putative 4-α-glucanotransferase and putative glycogen phosphorylase (7) are located with a 29 nucleotide overlap, likely constituting an operon on its genome. Since a debranching enzyme that mediates glycogen degradation is a member of the 4-α-glucanotransferases, we initially speculated that the enzyme encoded by the *malQ* gene may be involved in glycogen degradation as a debranching enzyme together with the glycogen phosphorylase encoded by the *glgP* gene.

However, we recently reanalyzed the genome sequence around the *glgP* gene region and found a potential promoter-like sequence located in the 114-bp intergenic region between the *malQ* and upstream *malR* genes. In addition, a MalR-binding consensus sequence reported in *Streptococcus pyogenes* (8) was detected between the putative *malQ* -35 and -10 sequences. We constructed a *malR* mutant in strain UA159. The phenotype of this mutant concerning GlgP expression was constitutive. Therefore, we concluded that the *malR* gene was the negative regulator of the putative *malQ/glgP* operon. In addition, this consensus sequence was also found in the promoter regions of the *malT* (symbolized as *ptsG* in the genome data) and *malX*FGK operons respectively encoding IIABCmaltose of the phosphoenolpyruvate-dependent maltose phosphotransferase system (PTS) (9) and the MalXFGK-binding protein-dependent ABC transporter for maltooligosaccharides (10, 11). Furthermore, the other glycogen phosphorylase gene *phsG* were located on another part of the chromosome (7) as a cluster with the glycogen synthesis genes (*glgBCDA*), although a candidate for a debranching enzyme was not present in this gene cluster.

A major carbon source during a ‘feast’ period in the human oral environment is dietary starch. However, *S. mutans* could not grow with starch as a direct carbon source, but grew well with maltose or maltooligosaccharides derived from starch in the presence of a small amount of saliva. Therefore, we presumed that the *malQ/glgP* genes may be involved in the catabolism of maltose or maltooligosaccharides derived from the starch supplied in food in the oral environment rather than the degradation of intracellular glycogen-like polysaccharides. This suggested that this organism, as well as other oral streptococci (12), indirectly utilizes starch in oral biofilms. However, the energy metabolism of starch-degradation products in *S. mutans* has not yet been fully elucidated. Therefore, we characterized the *malQ* and *glgP* genes in this study.

## Materials and methods

### Bacterial strains

*S. mutans* strains used were UA159 (7) and its mutants fkU1 (*malR*), zJU1 (*malQ*), zKU1 (*glgP*), and aeU1 (*malQ*, *glgP* ^*c*^). Streptococci were maintained on Todd-Hewitt (TH) broth/agar plates with or without appropriate antibiotics. *Escherichia coli* strain TOP10 was used as a host with the vector pBAD/HisA for the expression of cloned genes as N-terminal histidine-tagged proteins.

### PCR amplification of fragments to express or inactivate specific genes

**Fig. 1:**
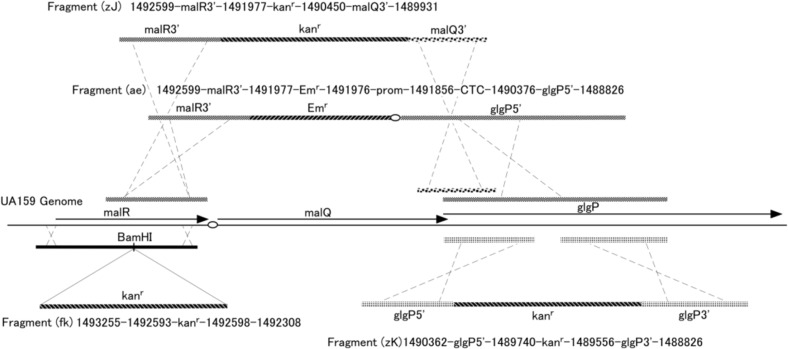
Construction of linear fragments for transforming *S. mutans*. Transformation was carried out as described previously (13). Numbers depicted above and below the fragments (fk), (zJ), (zK), and (ae) correspond to the nucleotide positions on the complementary strand of the UA159 genome. Ovals in the figure indicate promoter regions, which are abbreviated as ‘prom’ containing a 114-bp intergenic region between the *malR* and *malQ* genes. A MalR-binding consensus sequence is located between the -35 and -10 regions of a putative promoter sequence. CTC in the fragment (ae) are added nucleotides for splicing the fragments and are located immediately upstream from the Shine–Dalgarno sequence of the *glgP* gene.

The polymerase chain reaction (PCR) primers used in this study are listed in Table 1. All amplification reactions were carried out with high-fidelity DNA polymerase, KOD-Plus (Toyobo, Osaka, Japan) without terminal deoxynucleotidyl transferase activity. Regions corresponding to the *malQ* and *glgP* genes in strain UA159 were amplified with the primer sets exmalQ5/malQ32 and expglg51/expglg31, respectively. Amplified fragments were purified, digested with *Xho*I, and subcloned into *Xho*I/*Pvu*II-double-digested pBAD/HisA. The fragment (fk) was constructed by inserting the kanamycin-resistant gene cassette into the *Bam*HI site in the middle of the *malR* gene, which was subcloned into a pUC-vector (Fig. 1) (13). Splicing by the overlapping extension method (14) was employed to construct the linear fragments (zJ), (zK) and (ae) used to transform *S. mutans* UA159 resulting in *malQ*- and *glgP*-mutants (zJU1, zKU1 and aeU1). Target regions containing primer-annealing sites that were used in this technique are noted in Table 1 and the targeted fragments corresponded to those in Fig. 1, indicating spliced linear fragments for transforming *S. mutans* UA159.

**Table 1:** Primers used in this study

Primer designations	Sequences (5′>3′)	Purpose or target region
exmalQ5	ATCTCGAGATGGAAAAACGCGCAAGTGG	MalQ expression
malQ32	TGGACATACTTACTGAATTGTTTTGTTTTT	MalQ expression
expglg51	ATCTCGAGGAAATGAAGAAAATGACACAAAA	GlgP expression and glgP5′ in (ae)
expglg31	CTTTTTACAAACTATGCCAAATGTCC	GlgP expression
malR53	GGGATCCTGTACGTCAATATCTGA	malR3′ in (ae)
EmTailmalR3	CACACTCTTAAGCTAGCTTTTTTTAAGATTACGGACA	malR3′ in (ae)
malRTailEmF	TAAAAAAAGCTAGCTTAAGAGTGTGTTGATAGTGCAGT	Em^r^ in (ae)
IGTailEmR	AAGCTTCTAATCTCTAGGGACCTCTTTAGCTCCT	Em^r^ in (ae)
EmTailmalQ5	AGGTCCCTAGAGATTAGAAGCTTAATTTCTAAGCTTTT	Promoter region in (ae)
XhglgTailIG3	TTCTTCATTTCCTCGAGATTTTCCATATAGTCTCCTCGCT	Promoter region in (ae)
glg31	AACATAAAGAGCATTCATTTGCTG	glgP5′ in (ae) & glgP3′ in (zK)
expglg52	ATCTCGAGAATGACACAAAAAACAAAACAATTCAG	glgP5′ in (zK)
kanTailglg3	GGTTTATCCGGGATCCGTCAAGCCATAATCAAGACCATCA	glgP5′ in (zK)
kanF	GGATCCCGGATAAACCCAG	kan^r^ in (zK)
kanR	GCGGATCCCGAGCTTTT	kan^r^ in (zK)
kanTailglg5	AAGCTCGGGATCCGCTGGCTGATTATGCCTATGTG	glgP3′ in (zK)

Underlined sequences are those of added nucleotides for restriction endonuclease reactions or those necessary for the splicing by the overlapping extension method.

### Sample preparation, SDS-PAGE, and Western blot analysis

*S. mutans* strains and mutants were grown in 10-ml BTR-Sugar broth (15) (1% Bacto Tryptone, 0.1% Bacto yeast extract, 0.05% sodium thioglycolate, 0.61% K_2_HPO_4_, 0.2% KH_2_PO_4_, 1 mM MgSO_4_, 0.1 mM MnSO_4,_ 0.2% sugar). Cells were harvested, washed, disrupted with a Tissue Lyser (Qiagen, Hilden, Germany) with 0.2 mm diameter ceramic beads, and subjected to centrifugation (12,000×g, 5 min) with a microfuge to remove undisrupted cells. Four hundred microliters of supernatant fluid was obtained as a crude extract sample and its protein concentration was determined with DC protein assay reagents (BioRad, Hercules, CA, USA). SDS-PAGE was run with 2.5 µg of samples for Quick-CBB-PLUS (Wako Pure Chemical Industries, Osaka, Japan) staining and 0.4 µg of samples were used for Western blot analysis with previously prepared anti-GlgP serum (Operon Biotechnology, Tokyo, Japan) as described previously (16).

*E. coli* clones designated as ZF27 and ZF32 expressed the GlgP and MalQ proteins, respectively. The cells of these strains grown with 100-ml LB broth supplemented with 4×10^–3^% arabinose as an inducer were collected, washed, and subjected to 20 cycles of 30-second ultrasonication and 60-second incubation periods in an iced water container to obtain crude cell-free extracts.

The GlgP and MalQ proteins were purified with Ni-Sepharose 6 Fast Flow resin (GE Healthcare KK, Tokyo, Japan) as described previously, and the protein concentrations of the purified proteins were determined with DC protein assay reagents. Purified samples were frozen at −20°C for later enzyme assays. The enzyme activities of these samples were stable for at least 8 months.

### Enzyme assays for glucose-releasing and phosphorylase activities

Enzyme reactions both for the MalQ and GlgP proteins were performed in 50-µl reaction mixtures within a thin-wall-PCR tube with starch-degradation products as substrates. The standard enzyme reaction mixtures for glucose- and glucose-1-phosphate (G1P) assays were composed as recommended by the supplier (Oriental Yeast Co. Ltd, Tokyo, Japan) of enzymes used in the assay system. The enzyme reaction mixture contained 0.7–1.0 µg of purified MalQ protein or 2.5–3.2 µg of purified GlgP protein in addition to 50 mM potassium phosphate (pH 7), 0.5 mM MgCl_2_, and sugar substrate. The sugar substrates used in the enzyme reaction were 1% maltose, maltotriose, maltodextrin, and 0.8% amylodextrin (final concentrations). Both glucose-releasing and phosphorylase reactions were not linear as a function of time. Therefore, the incubation time was fixed at 10 min at 37°C followed by 5-min inactivation at 95°C. Glucose and G1P were formed in the reaction mixtures containing MalQ and GlgP proteins, respectively. Glucose or G1P in aliquots were spectrophotometrically determined by the end-point method of absorbance changes at 340 nm as a result of the amount of generated NADPH in the assay mixtures as recommended by the supplier (Oriental Yeast Co. Ltd) of the enzymes. Glucose and G1P assays were started with the addition of 2 IU of hexokinase and phosphoglucomutase, respectively.

### Monitoring for growth of UA159 and its specific mutants

The growth of *S. mutans* strains and mutants in BTR-sugar broth was measured at an optical density (OD) of 660 nm with the Ultrospec 500 pro spectrophotometer (GE Healthcare Life Sciences, Uppsala, Sweden). Values of OD_660__nm_ were recorded at 1-hour intervals following the inoculation of cultures into screw-capped glass tubes containing BTR-sugar broth. Sugars included maltose, maltotriose, maltodextrin, and amylodextrin as starch-degradation products, other disaccharides, as well as glucose as a control.

### Thin-layer chromatography

The reaction products liberated in the MalQ enzyme reaction mixture described above incubated with 1% maltose as a substrate for 100 min were characterized with respect to molecular size by thin-layer ascending chromatography on a 0.25-mm silica-gel-coated glass plate (TLC Silica Gel 60; Merck, Darmstadt, Germany) employing a solvent system of 1 M lactic acid/acetone/2-propanol (2: 4: 4, by vol.) (17). Carbohydrate spots were visualized by soaking the chromatogram into *p-*anisaldehyde solution containing ethanol/sulfuric acid/acetic acid/*p-*methoxybenzaldehyde (68: 2.5: 1.2: 1.9) and heated on a hotplate (Model 49SH, Fisher Scientific, Hampton, NH, USA) for 40 min at a dial setting of 4.5.

## Results

### Enzyme activities of the MalQ and GlgP proteins

The MalQ or GlgP protein was expressed as a His-tagged protein, purified, and the enzyme activities were determined as described in ‘Materials and methods’ section. The MalQ protein exhibited glucose-releasing activities with maltose and maltotriose and low activity with maltodextrin but not with amylodextrin. The GlgP protein did not exhibit phosphorylase activities with maltose and maltotriose but did with maltodextrin and amylodextrin (Table 2). Moreover, no glucose-releasing activity by the GlgP protein and no phosphorylase activity by the MalQ protein from any of these sugars were also confirmed.

**Table 2:** Enzyme activities of the MalQ and GlgP proteins

Purified MalQ (ZF32) protein				

Substrate	Maltose	Maltotriose	Maltodextrin	Amylodextrin
Glucose-releasing activities	88.7±27.1*	62.8±6.4	17.4±2.7	≈0 (IU)
Purified GlgP (ZF27) protein				
Phosphorylase activities	≈0	≈0	8.26±0.93	8.51±0.41 (IU)

* In order for absorbance changes (Δ*A*) at 340 nm to be 0.1–0.4, the volumes of aliquots from reaction mixtures to assay mixtures were adjusted. When Δ*A* was <0.02, it was regarded as no activity (≈0). Mean±SD with duplicated three independent experiments are given.

### Expression of the GlgP protein and construction of the malQ/glgP mutants

**Fig. 2:**
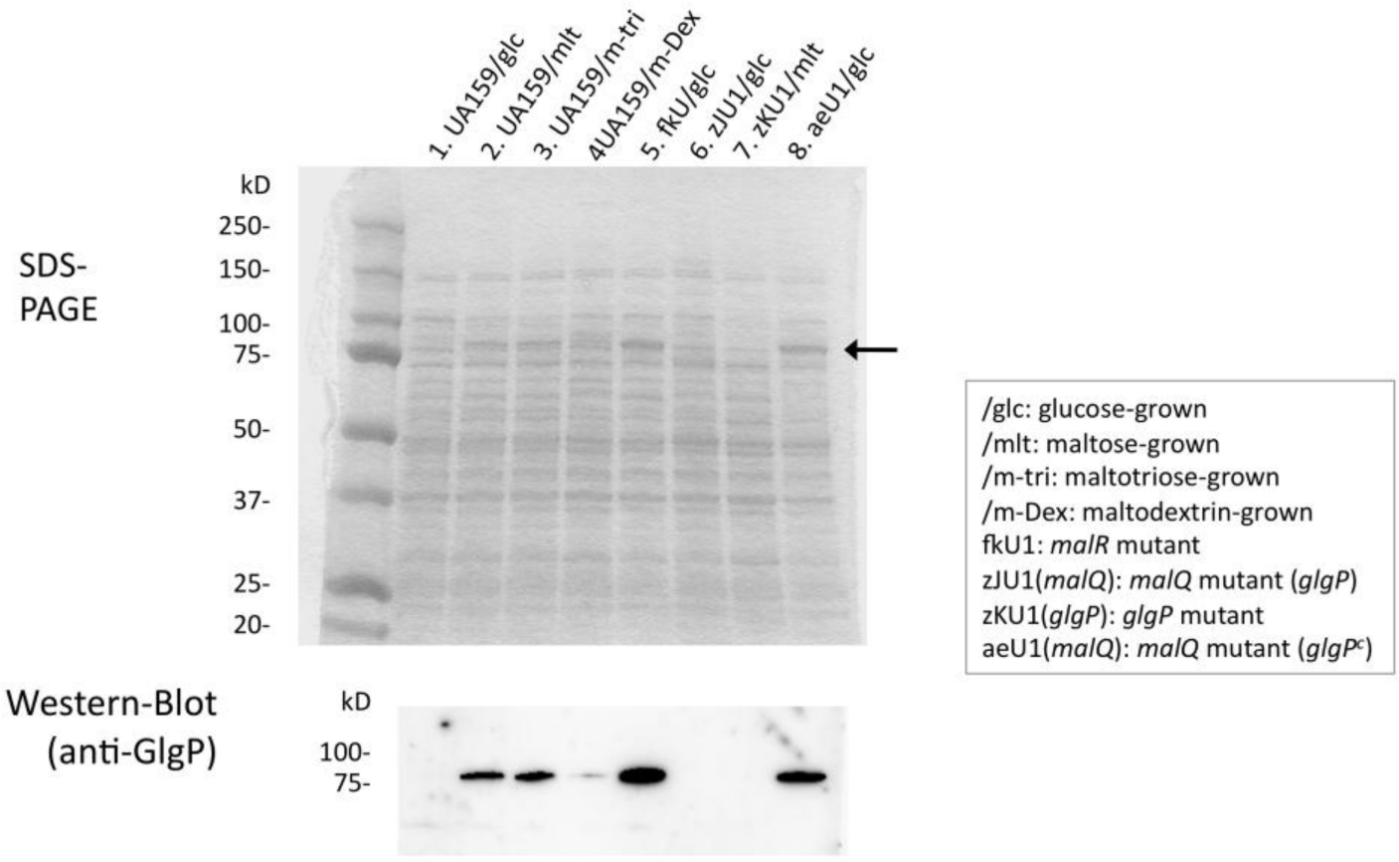
Expression of the GlgP protein in *S. mutans*. SDSPAGE and Western blot analysis were performed as described in the text. An arrow indicates GlgP protein bands appeared in the SDSPAGE. Cells were grown in BTR-sugar broth.

**Fig. 3:**
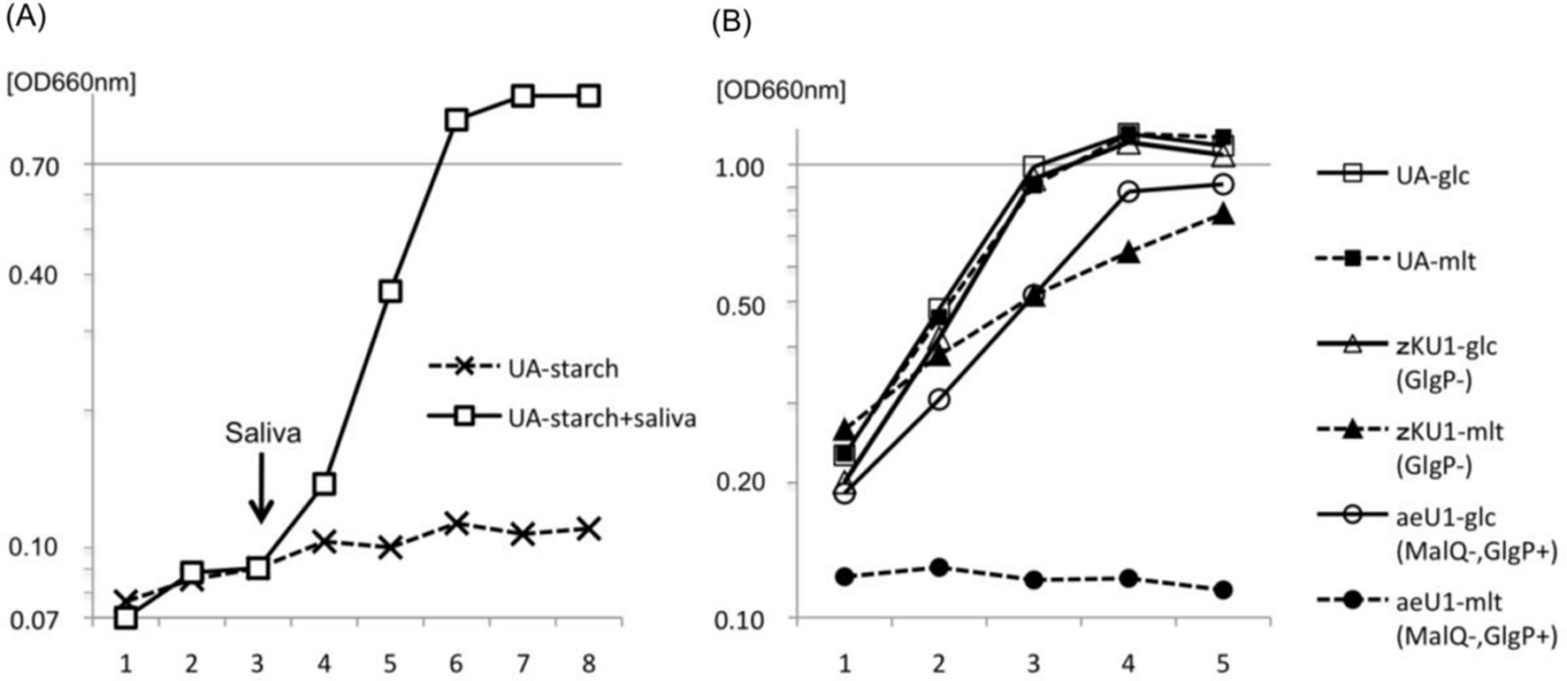
Growth of UA159, *malQ* mutant aeU1, and *glgP* mutant zKU1. The growth medium used was BTR-broth, whose composition is described in the text. Ten microliters of filter-sterilized saliva were added at the arrow into 10-ml culture of strain UA159 in panel (a). Abbreviations: UA-, UA159. The mutant aeU1 did not grow with maltotriose or maltodextrin and the mutant zJU1 also did not utilize these maltooligosaccharides as well as maltose. These results were eliminated from panel (b) of this figure to avoid ambiguity but are presented with the results for aeU1-mlt.

The GlgP protein was not induced in wild-type strain UA159 when grown with glucose but was markedly induced with maltose and maltotriose, which are starch-degradation products, as indicated by Western blot results (Fig. 2). GlgP protein bands were also observed with CBB (Coomassie Brilliant Blue) staining of the SDS gel (arrow in Fig. 2). Their expression levels were similar to those of glucose-grown *malR-*mutant fkU1 and aeU1 (Fig. 1), in contrast to no GlgP expression in *glgP*-mutant zKU1 and mutant zJU1 affected by the polar effect of the upstream *malQ* insertional inactivation. These results suggest that the MalR protein acted as a negative regulator of the *malQ*/*glgP* operon and that maltose and other starch-degradation products induced the transcription of this operon by inactivating the MalR protein. *S. mutans* was unable to ferment starch but able to grow with starch in the presence of saliva (Fig. 3a). Therefore, we hypothesized that the *malQ* and *glgP* genes are involved in the energy metabolism of starch-degradation products.

We initially constructed the mutant zJU1 in which the *malQ* gene was inactivated by replacement of the chromosomal DNA fragment encompassing a *malQ* upstream region and 93% of the *malQ* 5′ gene region with the kanamycin-resistant gene kan^r^ (Fig. 1). This mutant contained the intact *glgP* gene but did not express GlgP protein likely due to a polar effect on the *malQ*/*glgP* operon as suggested above. A characteristic phenotype of the mutant zJU1 was no growth with maltose as the sole carbon source. However, we could not determine whether the phenotype resulted from the absence of the MalQ or GlgP proteins in this mutant. Therefore, we constructed another *malQ* mutant in an attempt to be able to constitutively express the GlgP protein. The chromosomal DNA region around the *malQ* gene and the fragment (ae), which was used to transform *S. mutans* strain UA159, are also indicated in Fig. 1. The resultant MalQ-negative mutant was designated as aeU1. When we constructed the fragment (ae), we planned to use the segment malR3′ (Fig. 1) by employing the corresponding region from the chromosome of a strain harboring a mutation in the *malR* gene. We confirmed the constitutive expression of the GlgP protein with glucose-grown cells in contrast to the absence of GlgP expression in glucose-grown UA159 or zJU1 cells as indicated in Fig. 2. The GlgP-negative mutant zKU1 was constructed by the transformation of strain UA159 with the fragment (zK), in which a *glgP* internal fragment was replaced with the streptococcal kanamycin-resistant gene kan^r^ (Fig. 1).

### Growth of UA159 and its specific mutants, aeU1 and zKU1

Parental strain UA159 grew well with either glucose or maltose as a carbon source (Fig. 3b). In contrast, the *malQ* mutant aeU1 exhibited no growth with maltose. This mutant still utilized glucose, although the growth rate and yield of this mutant were slightly lower than those of strain UA159. No clear differences in growth rate and yield were observed between UA159 and aeU1 when melibiose, raffinose, lactose, trehalose, or sucrose was supplied as sole carbon sources (data not shown). The finding that this mutant could not grow with maltotriose or maltodextrin (data not shown) in addition to maltose suggests that the *malQ* gene is very likely essential for the utilization of starch-degradation products, but does not participate in the energy metabolism of the other sugars mentioned above. The GlgP mutant zKU1 grew well with glucose similar to UA159. However, the growth of this mutant in the presence of maltose as the sole carbon source was slightly less than that when glucose was used as a carbon source. The reason for this phenomenon will be discussed below. These results together with the observation that the GlgP protein had no phosphorylase activities for maltose or maltotriose described above suggest that the *glgP* gene is not essential for, but somewhat influences the utilization of maltose and maltotriose.

### Reciprocally additive effect on the enzyme activities of the MalQ and GlgP proteins

The *malQ* and *glgP* genes were likely co-transcribed because the *malQ* mutant zJU1 was GlgP negative as described above. Therefore, these two proteins may work together on their substrates or products *in vivo*. Glucose-releasing and phosphorylase activities on starch-degradation products were determined in the presence of both of the purified MalQ and GlgP proteins (Table 3). The most significant changes were the phosphorylase activities of GlgP proteins with maltose and maltotriose in the absence and presence of the MalQ protein. Glucose-releasing activities in the presence of the GlgP protein were almost unchanged from those without the GlgP protein.

**Table 3:** Reciprocally additive effect on the enzyme activities of the MalQ and GlgP proteins

Purified MalQ (ZF32) + GlgP (ZF27) proteins				

Substrate	Maltose	Maltotriose	Maltodextrin	Amylodextrin
Glucose-releasing activities*	100.1±21.1	70.2±14.4	20.91±1.3	≈0
Phosphorylase activities*	2.03±0.26	6.96±1.51	9.23±2.07	7.78±1.52 (IU)

* Both MalQ and GlgP proteins are contained in the reaction tube. However, glucose-releasing and phosphorylase activities were separately calculated for MalQ and GlgP proteins, respectively. Mean±SD with duplicated three independent experiments are given.

### The reaction products liberated with the MalQ enzyme reaction from maltose

**Fig. 4:**
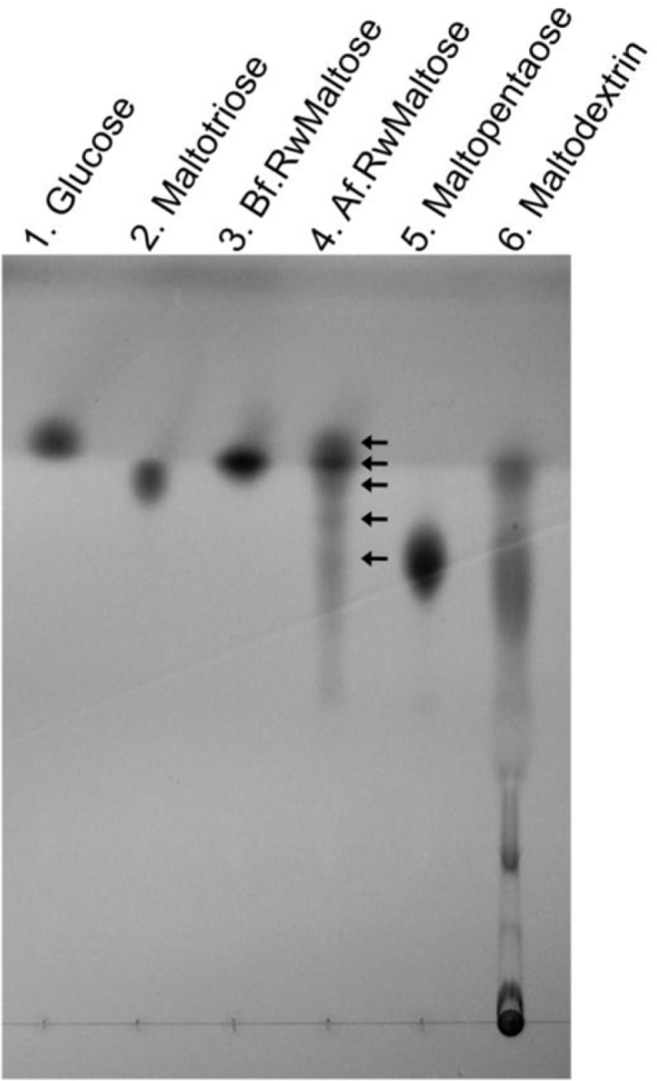
Thin-layer chromatography (TLC) analysis. An aliquot (2.5 µl) of the reaction mixture incubated for 100 min containing products from the activity of the purified MalQ enzyme was spotted at lane 4 next to the control (lane 3) spotted with a 1 µl aliquot of enzyme reaction mixture before incubation. Lanes: 1, 0.5 µl of 1% glucose; 2, 1 µl of 1% maltotriose; 5, 1 µl of 1% maltopentaose; 6, 1 µl of 1% maltodextrin. Arrows indicate glucose, maltose, maltotriose, maltotetraose, and maltopentaose. Abbreviations: Bf.RwMaltose, before reaction with maltose; Af.RwMaltose, after reaction with maltose.

The MalQ enzyme assay to examine the reaction products released from maltose was performed as described above (Tables 1 and 3) except for the extended reaction time (100 min). Thin-layer chromatography (TLC) indicated that maltooligosaccharides including maltotriose, maltotetraose, maltopentaose and higher molecular oligomers as well as glucose were generated in the reactions (Fig. 4, lane 4). This suggests that the MalQ protein does not encode α-1,4-glucosidase activity but is a 4-α-glucanotransferase.

## Discussion

4-α-Glucanotransferase was first reported in potato tubers in the 1950s as a disproportionating enzyme with distinct activity from the glycogen debranching enzyme, and genes encoding this enzyme activity were characterized later among *Streptococcus pneumoniae* (*malM*) (18), *Clostridium butyricum* (*malQ*) (17), and *E. coli* (*malQ*) (19) as well as potato (*malQ*) (20). 4-α-Glucanotransferase in the above organisms catalyzes a reaction in which single or multiple glucose units from the non-reducing ends of maltooligosaccharides are transferred to the 4-hydroxyl group of acceptor sugars. Glucose and maltose act only as acceptors, whereas maltotriose is the smallest donor substrate for these enzymes (17, 19) (20). Therefore, these enzymes do not release glucose from maltose, but release it from maltodextrin or amylodextrin. In contrast, the *S. mutans* MalQ enzyme released glucose from maltose (Table 2). How could the *S. mutans* MalQ enzyme release glucose from maltose? One possibility is that this enzyme may be able to use maltose as a donor molecule. Medda reported amylomaltase (4-α-glucanotransferase) activity in partially purified preparations obtained from *S. mutans* strain 6715-49 (this strain is currently classified as *S. sobrinus*) cells grown with maltose as the main carbon source, and indicated that maltose participated as a donor as well as an acceptor for the enzyme activity (21). *S. mutans* MalQ reaction products were not only glucose but also maltotriose and higher molecular weight maltooligosaccharides as shown in Fig. 4, and these products were derived initially from maltose. Therefore, maltose participated as a donor of the reaction mediated by the MalQ enzyme likely to be a homolog of the *S. sobrinus* 4-α-glucanotransferase enzyme.

We demonstrated the glucose-releasing activity of the MalQ protein from maltose and maltotriose. However, the physiological substrate of this protein in *S. mutans* cells may be maltose-6-phosphate, which is taken up into cells predominantly through IIABCmaltose (the *malT* product) of the phosphoenolpyruvate-dependent maltose PTS in *S. mutans* (9). In this respect, Mokhtari *et al*. recently reported a novel maltose-6-phosphate phosphatase (MapP) in *Enterococcus faecalis* (22). The *mapP* gene was located downstream from the enterococcal *malT* gene encoding a maltose-specific EIICBA of the PTS, and was previously suggested to encode an endonuclease/exonuclease/phosphatase family protein of unknown function. The *S. mutans malT* gene was followed by a gene locus tagged as ‘SMU_2046c’, which encoded the same family protein suggested by the conserved domain search of the BLAST program. Therefore, SMU_2046c is very likely the *mapP* homolog, although this remains to be confirmed. Accordingly, the MalQ enzyme of *S. mutans* likely utilizes maltose as a substrate.

The *malQ* mutant aeU1 exhibited no growth with maltose but still utilized other disaccharides including sucrose, lactose, trehalose, and melibiose as described above, which is consistent with the finding that *S. mutans* possesses transporter and sugar (phosphate) hydrolase protein pairs specific for these disaccharides that induce the corresponding pairs (23–27). In contrast, the *malT* gene encoding the maltose transporter (IIABC) of PTS was not proximal to the corresponding sugar phosphate hydrolase gene, which may be compatible with a *malT*-*mapP* gene arrangement in *E. faecalis*.

Even if such a hydrolase for maltose is absent in *S. mutans* cells, starch-degradation products including maltose as the smallest molecule would be catabolized in concert with the *malQ*, *glgP*, and *amy* (28) gene products. The GlgP protein did not phosphorylize maltotriose or maltose, but phosphorylized saccharides larger than maltodextrin as a substrate (Table 2). However, this enzyme apparently catabolized maltotriose or maltose (Table 3) in the presence of the MalQ protein. This did not appear to result from changes in the substrate specificities of the GlgP protein but may have resulted from the generation of maltooligosaccharides and glucose from maltose and maltotriose mediated by MalQ enzyme reactions (Fig. 4). The growth of the *glgP* mutant zKU1 was suppressed when maltose was used as the sole carbon source (Fig. 3b). This may be explained by the intracellular over-accumulation of maltooligosaccharides mediated by the MalQ glucanotransferase activity in the absence of GlgP phosphorylase activities.

*S. mutans* alone was unable to ferment starch (Fig. 3a) similar to other oral streptococci (29). However, oral biofilm bacteria as well as tooth hydroxyapatite actively bind salivary amylase (30), which degrades starch to maltose, maltotriose, and maltodextrin fermentable by *S. mutans*.

We now report that the purified MalQ and GlgP proteins exhibited glucose release from maltose/maltotriose and phosphorylase activity from maltodextrin/amylodextrin, respectively. In addition, the *malQ* gene involved in glucose-releasing activity is essential for utilizing starch-degradation products similar to the *malP* maltose phosphorylase gene in *E. faecalis* (22). Therefore, the *malQ* gene may be a target for controlling *S. mutans* in oral environments.
